# The family dynamics of children on the streets of Ibadan, Southwest Nigeria

**DOI:** 10.4102/safp.v65i1.5774

**Published:** 2023-12-14

**Authors:** Abimbola M. Obimakinde, Moosa Shabir

**Affiliations:** 1 Family Medicine Unit, Community Medicine Department, Faculty of Clinical Sciences and Faculty of Public Health, College of Medicine, University of Ibadan, Ibadan, Nigeria; 2 Department of Family Medicine, University College Hospital, Ibadan, Nigeria; 3 Department of Family Medicine and Primary Care, School of Clinical Medicine, Faculty of Health Sciences, University of the Witwatersrand, Johannesburg, South Africa

**Keywords:** family dynamics, streetism, hawking, children, interpersonal relationship, SCREEM, APGAR, Ibadan

## Abstract

**Background:**

Children roaming the streets estimated at 1 in 10 by a 2021 United Nation Children’s Funds (UNICEF) report is a growing problem, in cities of lower- and middle-income African countries. Studies of street children with no family ties abound, but there is a paucity of studies on children on the street who exist within families and return home daily. We explored the family dynamics of children on the streets of Ibadan, emphasising family structure, resources and relationships.

**Methods:**

Using an exploratory design based on a qualitative approach 53 participants were interviewed, including children on the streets, parental figures, child-welfare officers and street shop owners. Participants were selected from streets in the five urban local government areas of Ibadan, Nigeria. Recorded data were transcribed, and framework analysis was performed.

**Results:**

The family dynamics included family structural problems, poor family resources and poor parent-child relationships. The family structural problems included: broken homes, large families and ambivalence around polygamy as subthemes. Family resources comprised: poor economic resources, poor social resources, educational challenges, cultural ambivalence and spiritual backdrops. The family relationships patterns included: poor adaptability, economic-oriented partnership, poor growth support, poor emotional connection and poor family bonding.

**Conclusion:**

The dynamics driving a family’s choice for child streetism in Ibadan, mostly to hawk, are devaluation of family life, parenting irresponsibility, and poor filial relationship, underscored by economic constraints and socio-cultural decadence. The results of this research buttress the need for family-level interventions to forestall the escalating phenomenon of child streetism in Ibadan, Nigeria.

**Contribution:**

This research highlights the family dynamics of children on the streets, and buttresses family-level interventions are necessary to forestall escalating child-streetism in Ibadan, Nigeria.

## Introduction

‘Child-streetism’ describes the presence of children below the age of 18 years who for various reasons are seen on the street without any apparent adult supervision.^1^ There are four categories of street children: children-of-the-streets with severed families link, children-from-street-families, children-who-absconded-from-institutional care and children-on-the street.^2,3^ The children-on-the-street is the dominant category of street children.^2,3^ The children-on-the street are distinctly different, as they spend time on the streets during the day but return home daily to their families at night. They constitute 80% – 90% of street children in urban regions of low- and middle-income countries (LMICs), like Nigeria.^2,3,4^ There is an estimated 15 million street children in Nigeria; and intriguing is the fact that the highest proportion are those who have links to their families, which suggests family systems failure.^3,5^ A week-long survey conducted in the year 2021 on the streets of Ibadan, Nigeria, revealed 5026 children most of whom come daily from a home.^6^ An estimated 100 000 children with existing family ties were also found on streets of Lagos, Nigeria, in 2022.^6^ Lagos is proximate to Ibadan in Southwest Nigeria, with similar demography and socio-economic characteristics.

Family dynamics refers to the family structure and the pattern of relationship and support among the family members.^7^ Each family member has a role to play that shapes family dynamics.^7^ Inability of members to play the expected roles can lead to family dysfunction with repercussions for the welfare of children in the family.^7^ Parental role is that of a provider and a nurturer with the child at the receiving end. An atypical family composition, inadequate resources and interactions can push children to the street.^8,9,10^ The 21st-century LMICs are witnessing changes in family dynamics and consequent poor child outcomes in the background of socio-economic challenges.^3,5^ In Nigeria, seemingly, the prevailing socio-economic realities are influencing family dynamics and the continuing phenomenon of street children. However, the majority of publications addressed children of the streets who are completely detached from their families with poor specificity regarding children on the street.^2,3,8,9^

The socio-economic realities include urban slum unstructured families, with one or both parents earning below minimum wage and desertion of the family by at least one parent.^8,9,10^ The minimum wage in Nigeria is approximately $39.00 monthly and insufficient to cater for a family’s needs.^11^ The unemployment syndrome and rising inflation engender relocation of parents within the country with eventual desertion of the children.^11,12^ Poor population control with economic hardship in families,^11,12^ might also be contributing to streetism for family-connected children but evidences are lacking in current literature.

The situations of street children with severed families can be a logical extension of the family failure but the drivers of streetism for a child existing within a family need deciphering. Several processes within the family can influence how members relate to each other, perform their roles and transit through expected and unexpected life events.^7,13^ The filial (parent–child) relationship in the family system, comprising adaptability, role performance, and communication, is important and can propel child streetism when distorted.^14^ It seems families of children on the street are unable to adapt to their life situations and this engenders the unusual ecological crossing to the street. However, there are queries regarding the differences between the family dynamics of children-of-the-street and children-on-the-street. A meta-analysis and other studies across the world reported that street children are a by-product of socio-economically challenged families who are sent out of their homes to provide financial sustenance for themselves.^2,3,5,10^ The aforementioned studies spoke extensively about children of the street. For instance, family disintegration and poor parent–child interactions have been reported as correlates of child-streetism but most publications speak to the minority categories of street children.^3,5^ Extensive research has been conducted mostly on children of the street in West Africa and Nigeria, especially the Almajiris in Northern Nigeria.^2,8,9^ There are gaps in the literature regarding the family dynamics of children-on-the-streets which this research hopes to fill, focusing on children on the streets of Ibadan, the largest metropolis in southwest Nigeria with escalating child streetism phenomena. Our objectives were to explore the family structure, family resources and filial relationships of children on the streets of Ibadan.

## Conceptual framework

Family resources and interpersonal relationships are the family dynamics relevant to the context of children on the street phenomena. Therefore, the family SCREEM and APGAR^15,16^ were apt for the conceptual framework and observational tools of the research. Gabriel Smilkstein adopted the SCREEM acronyms for the social, cultural, religious or spiritual, economic, educational, and medical domains, describing the various resources available within a family that may support a child or any other family members.^15,16^ The family SCREEM aids the understanding of resources that constitute the family’s abilities and weaknesses.^15,16^ The family APGAR, also created by Smilkstein, is an acronym for adaptation, partnership, growth, affection, and resolve in a family.^15,16^ The APGAR is a measure of family functioning, which assesses five components of the interpersonal relationship between any family members,^15,16^ and is applicable to filial relationships.

The SCREEM can be considered flawed because religious and spiritual were interchanged despite the different meaning. However, both SCREEM and APGAR have good validity and cross-cultural applicability, as reported in a Nigerian study and other studies.^15,16,17,18,19^ The components of both APGAR and SCREEM were utilised as prompts during interview with our research participants. The SCREEM guided the exploration of the resources within the family while the APGAR guided the understanding of the relationship between the parental figures and the children on the streets of Ibadan. In addition, the family structure was assessed using the family composition, family size, parents’ availability, parents’ marital status, and parents’ involvement with child-rearing.

## Methods

### Research design

This qualitative research utilised in-depth interviews (IDI) with four groups of participants.

A qualitative approach afforded a rich exploration of the research objectives and provided vast unstructured open-ended data. These groups included: child welfare officers, street-shops owners, children on the street, and dyads of a parental figure and a child on the street of Ibadan, Oyo State. The diversity of participants was to ensure a multidimensional understanding of family dynamics of children on the street by sourcing information from the children and relevant adults within their ecosystem. These four sets of interviews provided an in-depth exploration of the family dynamics of children on the streets of Ibadan, Nigeria, using triangulated information from multiple sources.

### Sampling and participants

The study setting was five streets, one from each of the five urban local government areas (LGAs) of Ibadan, the second largest city in Nigeria and the capital of Oyo State, South-West Nigeria. The LGAs are: IDO, South-West (SW), North (N), North-East (NE) and North-West (NW). All participants were initially purposively selected and subsequently by snowball technique. Data saturation was achieved at a sample size of 10 per each category of participants.

The child welfare officers were Oyo State-appointed social workers. The social workers were selected at two per LGA and two from the central directorate for child welfare services of the Oyo state secretariat in Ibadan. The child welfare officers had worked at each LGA for a minimum of 6 months, and all were university graduates and many of them had a Master’s degree in social work. The selection of each street was guided by information from the child welfare department in each LGA. The streets selected were those with known active population of children on the streets.

Two street shops or street-business owners per LGA, who had been operating a shop or business for more than 6 months on each street were selected. Being a past child on the street was a desirable selection criterion, met by a few, which afforded a cognizant recount of their past history as a child on the street. The selected shop owners had shops location which granted them direct street views and were familiar with the children’s movement, activities and parental figures or family members. Therefore, they served as links to the parental figures, and assisted with the recruitment of the children on the streets. This research defined the family as a group of people that are biologically related.

A minimum of two children on the street were selected from each of the five urban LGAs after their parents’ permission was obtained. The children aged 13 years to 17 years and 11 months were individually interviewed; this age group selection was to allow for cognizance. Another set of two children per each LGA were selected and this second set of children were paired with a parent figure for paired interviews which were separately conducted.

### Data collection and procedure

Data were collected between June 2021 and September 2021 ensuring universal precautions against coronavirus disease 2019 (COVID-19). Research documents were written in a simple English language and the Yoruba (local) translation was available.

With the children’s knowledge, the research team approached and distributed consent forms to parental figures at work or home (distanced 20 min – 60 min drive from the streets). The children’s assent was obtained after parental permission. The parental figure or child decided the venue and time for the child’s interview. Each interview session was conducted by the first author with consideration of participants’ preferences and privacy. The interviews were moderated using an interview guide which included the five items of the Family APGAR as a probe for questions for exploring the interpersonal relationship between a child on the street and a parental figure. The six items Family SCREEM was used to probe the interview segment on characteristics of the families of children on the street. The interviews with the participants were audio-recorded using a digital recorder with written informed consent and/or assent. Safety and anonymity of the recordings were maintained by serial tagging and in safekeeping by the first author.

### Data analysis

Direct and complete transcripts of the audio-recorded interview sessions were translated into English and typed in Microsoft Word. The translators were trained research assistants with Masters in Public Health, and the accuracy of translation was ascertained by random cross-checking. Both authors independently read the transcripts and agreed on the themes. Framework analysis of the data was conducted by a systematic process and interpretation of data through five levels of processing including; familiarisation, identification of thematic framework, indexing, charting and mapping.^20^ The ATLAS.ti version 8.4 (ATLAS.ti Scientific Software Development GmbH, https://atlasti.com) qualitative data analysis software package was used for deductive and inductive coding with quotations specific to each theme and subtheme generated.

### Ethical considerations

Ethical clearance to conduct this study was obtained from the University of the Witwatersrand, Human Research Ethics Committee (No. M210424), and the Oyo State’s (Nigeria) Ministry of Health, Department of Planning, Research & Statistics (No. AD/13/479/4118A). Written informed consent and/or assent (where relevant) was obtained from each participant for the interview.

## Results

A total of 53 interviews were conducted as shown in Table 1. Themes and subthemes identified in the research are as shown in Table 2.

**TABLE 1:** Characteristics of participants.

Study sites	Child	Age(years)	Gender	Paired parent	Age(years)	Gender	Paired child	Age(years)	Gender	Shop owner	Age(years)	Gender	Welfare officer	Age(years)	Gender
Ido	C1	17	M	PP1	42	Mother	PC1	16	M	SO1	56	F	WO1	43	F
Ido	C2	14	M	PP2	40	Father	PC2	15	M	SO2†	35	M	WO2	52	F
SW	C3	17+	F	PP3	35	Mother	PC3	13	M	SO3†	35	F	WO3	41	F
SW	C4	17	F	PP4	30	Mother	PC4	17	M	SO4†	40	F	WO4	55	F
N	C5	15	M	PP5	30	Mother	PC5	17+	M	SO5	32	F	WO5	46	F
N	C6	14	F	PP6	52	Mother	PC6	16	M	SO6	30	M	WO6	54	F
NE	C7	15	M	PP7	42	Mother	PC7	17	M	SO7†	41	F	WO7	41	F
NE	C8	17	F	PP8	38	Mother	PC8	15	M	SO8	28	M	WO8	43	M
NW	C9	17+	M	PP9	40	Mother	PC9	17+	M	SO9	45	F	WO9	56	F
NW	C10	17	M	PP10	60	Grandmother	PC10	13	M	SO10†	34	M	WO10	52	M
NW	C11	17	M	-	-	-	-	-	-	-	-	-	-	-	-
SS	-	-	-	-	-	-	-	-	-	-	-	-	WO11	37	F
SS	-	-	-	-	-	-	-	-	-	-	-	-	WO12	51	M

**Total**	**11**	**-**	**-**	**10**	**-**	**-**	**10**	**-**	**-**	**10**	**-**	**-**	**12**	**-**	**53**

SS, State Secretariat; M, male; F, female; C, child; PP, paired parent; PC, paired child; SO, shop owner; WO, welfare officer; SW, South-West; N, North; NE, North-East; NW, North-West.

† , Shop owner, who was previously a child on the street.

**TABLE 2:** Themes and subthemes from analysis.

Themes	Subthemes
1. Family structure	1.1 Broken homes 1.2 Large family size 1.3 Ambivalence around polygamy
2. Family resources	2.1 Poor economic resources 2.2 Poor social resources 2.3 Educational challenges 2.4 Cultural ambivalence 2.5 Spiritual backdrop
3. Family interpersonal relationship	3.1 Poor adaptability 3.2 Economic partnership 3.3 Poor growth support 3.4 Poor emotional connections 3.5 Poor family bonding time.

The family structure informed family resources, the strongest theme, which in turn was underscored by family relationships.

### Theme 1: Family structure

Three subthemes explained the family structure.

#### Subtheme 1.1: Broken homes

Most respondents shared that children on the street had typical family structures but with a fairly strong element of broken homes. Broken home in this research refers to a family in which the parents are separated for any reason. Children shared stories of fathers and mothers who had left their spouses for another partner or a situation where the father had to move to another part of the country for work. Mothers shared stories of challenging relationships with their ex-spouses, sometimes casual informal relationships or marital affairs that produced children. Some children had mothers or fathers that died, usually when they were younger, leaving them with a lone parent who is heavily reliant on grandparents:

‘They are from broken home raised by single mothers, and single fathers.’ (WO10, 52, M)

‘He [*father*] married another person … when my mummy died, he left us.’ (C6, 14, F)

‘I have a maternal grandmother and a paternal grandmother. I have a mother. It is only my father that is late.’ (PC10, 13, M)

‘My mummy and daddy were separated but my mummy was the first wife, when my daddy died things scattered.’ (C8, 17, F)

Shop owners and welfare officers shared similar stories of children on the streets from broken homes and instances of the child-care burden on grandparents and other relatives who use the children to supplement income for their household. Welfare officers and shop owners opined unresolved parental conflict leads to easily broken homes particularly for parents who were from broken homes themselves. They shared stories of fathers that were in other cities while their children lived with mothers or grandparents who were not caring enough. Welfare officers strongly opined that absentee parenting of these children leads to poor monitoring and their presence on the street:

‘Some mothers are separated from the child’s father, and the child is staying with a guardian who doesn’t have much money and therefore sends the child hawking.’ (SO1, 56, F)

‘The mother dropped the children with relatives and remarried another man.’ (WO2, 52, F)

#### Subtheme 1.2: Large family size

The children on the street came from families where there are more than four children. Sometimes being a female among males was seen as a role to support a mother working. Sometimes being a male with younger siblings was seen as a rationale to support the family:

‘My father has one wife, my mum, and eight of us children.’ (C3, 17, F)

‘My dad has one wife, and my mother has one husband and six children.’ (C7, 15, M)

Welfare officers, and some shop owners, bemoaned that families had many children without planning to give them the care needed. They suggested better family planning:

‘The proper thing is that parents right from day one of their marriage know their economic capability and how many children they can take care of.’ (WO12, 51, M)

‘There was a woman that birthed 16 children and she was selling groundnut and *garri* [*cassava flakes*]. The husband works as a gardener. The 16 children shared clothes because the parents can’t cater for them.’ (WO9, 56, F)

Not all children in a large family were on the street or encouraged to hawk. Many shared stories of smaller siblings that were at home usually with grandmothers and older siblings who were married, studying, or working and sometimes living in another part of the country. The older sibling would sometimes provide money for the family, but it would not be deemed enough:

‘My younger siblings are at home because they are not yet matured to hawk.’ (C7, 15, M)

‘My older sibling can support me but she is just learning a trade and not yet established.’ (C9, 17, M)

#### Subtheme 1.3: Ambivalence around polygamy

Most respondents shared that these children had monogamous parental structures although some shared polygamous parental structures. Polygamous marriages varied between conflictual and harmonious. Some shop owners and welfare officers had very strong views on polygamous marriages as a reason for children on the street; they said the rivalry between the wives affects the care given to the children by the fathers:

‘The father has a second wife and she does not allow him to pay attention to me [*first wife*] and my children.’ (PP7, 42, Mother)

‘I discovered that most of them come from a polygamous family and there is no way, a child from a polygamous family will be raised well, which was my story.’ (S010, 34, M)

‘Polygamous homes too can contribute, but it is not a common denominator.’ (WO3, 41, F)

Some welfare officers and shop owners were of the views that polygamy was not as important a factor as negligent parenting:

‘Some men with many wives will raise the children well and some with a single wife won’t. It is a matter of responsible parenting.’ (WO7, 41, F)

‘Even if they are many wives in the family, the wives could raise their children well.’ (SO4, 40, F)

### Theme 2: Family resources

This comprised of five subthemes including poor economic and social resources as main subthemes.

#### Subtheme 2.1: Poor economic resources

Poor economic resources were characterised by either unemployed parents or those employed in low-paying roles. There were instances of one parent with low-paying job (e.g., gatemen, farmer, shoe markers) and the other parent being unemployed which pushed the child on the street. The majority of the parents of the interviewed children are very small-scale traders, farmers, food vendors, and low-cadre office or factory workers. Shop owners and welfare officers opined that some parents are not gainfully employed:

‘His mother is currently not working.’ (PP10, 60, Grandmother)

‘Don’t mind him, his father is not working.’ (PP5, 30, Mother)

‘Those parents are jobless, they put any petty goods on a tray and tell the children to go hawk and make money before they can eat even breakfast.’ (SO8, 28, M)

‘Mostly, these children are those whose parents don’t have good jobs.’ (WO3, 41 years, F)

‘Mother is a trader, selling a variety of things, corn, pepper, vegetables, etcetera. My father is a farmer.’ (C3, 17, F)

Child welfare officers described 70% – 80% of the parents as the ‘poorest of the poor’ as quoted below:

‘What we mean by this are parents that could not feed themselves and their children three times a day, they struggle to eat once a day.’ (WO8, 43, M)

‘We went to forty communities whereby the fathers or mothers would send their children to the street to work before the family can afford a meal a day.’ (WO7, 41, F)

The shop owners and the child welfare officers explained the context of ‘hawking’. They reported the children were not willingly hawking, not forced into child labour nor growing their own business. They said the children ventured to the streets to fulfil their pressing needs because of the family’s financial hardship. They reported that children either beg for money, or plead to collect goods from shop owners to hawk without any down payment with hope of monetary gains from profits. It was gathered from some children that most parental figures were originally unaware of their street activities and when parents realised they are reluctant to call the children off the streets because of the monetary gains to the family.

#### Subtheme 2.2: Poor social support

Many respondents gave examples of a lack of spousal social support for the mothers, worse for unwedded mothers, wives in a polygamous marriage and parents living separately. The mothers are reportedly the available parental figures and are usually deprived of support from the fathers who are mostly inaccessible:

‘The ease of raising a child for the mother is smoother if the father is contributing. If their father is someone that supports me, the responsibilities won’t be heavy, for me.’ (PP5, 30, Mother)

‘He left me for another woman. When I call him once he hears my voice, he disconnects the call. The children have stopped calling him because he had severally rained curses on them.’ (PP3, 35, Mother)

Child welfare officers reported cases of unintentionally separated parents which degenerated into poor social support for mother and child. A couple of welfare officers and shop owners gave examples of socially inept immature parents:

‘The father lived and worked in Abuja, comes home after four months and the mother goes to the market daily leaving the children at home unattended and these children go hanging around the streets.’ (WO12, 51, M)

‘Most of these parents are “babies” when starting raising these children. They don’t have experience with parenting, they just observe and copy their neighbours’ attitude towards child rearing’ (SO7, 41, F)

#### Subtheme 2.3: Educational challenges

Many of the interviewed children and their mothers shared a strong value for schooling. All of the interviewed children were in secondary school or had just completed senior secondary school and anticipated further education. Respondents said street hustle for children serves as educational resources when parents are financially incapable:

‘Some children hawk after school to assist parents buy needed school materials. Some start hawking in the morning and do not go to school when their parents cannot afford school fees.’ (SO1, 56, F)

‘We have some children that are determined to go to school and the only option given to such, by the parents is to hawk to augment what the parents are earning.’ (WO11, 37, F)

‘For my WAEC [*West Africa Examination Council*] exams, I had to hustle for ten thousand naira, my parents struggled to add the rest of the money needed.’ (C1, 17, M)

The children often skip school and are found all day on the streets when school is not in session (holidays, midterm breaks or when exams are over even before the holidays). The children also skip school during school sessions or defer a whole academic calendar when they need to make money to cater for their school needs.

Most respondents reported that parental illiteracy and poor resources for the child’s education are major challenges and causes of the continuing presence of children on the street. A few respondents mentioned that some parents pull children struggling with academics out of school and send them to the street to make money for the family instead of investing money to assist the child’s learning ability:

‘The low standard of education of their parents leads to poor understanding of the necessity of education for some children. Some parents prefer children hawk or beg for their daily needs on the streets.’ (WO9, 56, F)

‘It was a year before my daughter would finish secondary school she got pregnant. She didn’t complete her education.’ (PP10, 60, Grandmother)

‘He wants to further his education but I think there is no financial means of making that happen, I won’t lie.’ (PP9, 40, Mother)

#### Subtheme 2.4: Cultural ambivalence

Culture is defined as a way of life and there were some interesting findings in this study. A few parents said child streetism is a culturally acceptable avenue for a child to understudy the family’s trade and acquire trading skills:

‘It is for him to know how to trade, that is why he hawks the meat I sell. I grew up following my mum to her meat stall and learned the business, so when I resigned from my police job, I easily could go back to being a butcher because I had gained the experience as a child.’ (PP2, 40, Father)

Welfare officers and street shop-owners inferred some Ibadan natives anecdotally disregard family life in favour of promiscuity, resulting in children who are uncared for, roaming the street:

‘Ibadan indigenes generally don’t care about keeping a home. They say any Ibadan indigene that does not engage in extramarital affairs is a bastard. The mother sends her children from different unions to live with her ex-husband’s mothers or her mother and the children are sent to the streets to fend for themselves.’ (CW5, 46, F)

A few child welfare officers spoke extensively about the loss of collectivist culture and culturally brainwashed illiterate wives, who birth unplanned children to satisfy their husbands’ desires:

‘Gone are the days when a child found to be wayward will be scolded by people in the neighbourhood.’ (WO11, 37, F)

‘The mother will say “I don’t want my husband to do anyhow or sleep around, let me just be giving him children.”’ (WO7, 41, F)

#### Subtheme 2.5: Spiritual backdrop

Respondents reported that some parents believe God will sort out their children when they are on the streets and some children share this belief with their parents:

‘Most of these parents don’t care to plan their family size, because they believe that God will take care of them. They are less concerned and so after a few months they are pregnant again.’ (WO3, 41, F)

‘I know destiny cannot be altered, God has destined that I will hawk on the street, and I have accepted my fate, that is how God has ordained it.’ (C4, 17, F0 [*7th of eight children in a monogamous union*])

An unemployed mother of six children (PP8, 38 years) whose first child (PC8, 15, M) carries loads on the street, claimed the reason for birthing up to six children was because her pastor advised her continuing birthing until the ‘rebirth’ of her second child who was a seer and died because of spiritual warfare.

We identified spiritual but no religious resource in the response from the research participants.

### Theme 3: Family interpersonal relationship

Interpersonal relationships, between the child on the street and parental figure, are strongly influenced by family resources and family structure. Family interpersonal relationships had five sub-themes.

#### Subtheme 3.1: Poor adaptability

Shop owners and child welfare officers opined that children on the street handle their problems by themselves with no effort from their parental figures. Respondents said children on the street living with guardians are more disadvantaged in this regard:

‘Child and parent don’t even communicate on personal issues; the mother does not even know how to ask “How was your day” and the child won’t have the chance to say “Mummy this happened on the street today.”’ (SO7, 41, F)

‘My uncle’s wife is not the kind of person someone can talk to, because she will not listen, even my uncle is not approachable.’ (C6, 14, F)

Shop owners who were previously children on the street shared stories of their guardians’ inability to assist them in handling troubling matters; but few children claim they occasionally discuss troubling matters with their mothers who are relatively more accessible:

‘I have been sorting myself out since I was 15 years, that time when we say “Grandma we are suffering” she will say “All is well”. Because she isn’t someone that has the power to fight for us so she can’t say “Come let me ease your troubles”.’ (SO10, 34, M)

#### Subtheme 3.2: Economic partnership

Child welfare officers and shop owners opined most of these children do not enjoy nurturing from the parental figure, but rather the children view themselves as financial partners and therefore do not see the need to share decision-making with parents:

‘The mother will go her way to make money and the child will go her or his way to hawk and they will all meet at home at night.’ (WO7, 41, F)

‘There are some children that once they start making money, the parents are unable to talk to or control such a child because the child sees himself or herself as breadwinner and treats the parents as mates.’ (SO5, 32, F)

A few children claimed they enjoy shared decision-making and good nurturing from their mothers but gave no specific examples, while many children reported the reverse:

‘I eat at home occasionally. It’s not that I don’t like the food but because I have money, I want to buy what I desire. At times if mother tells me to wash plates, I reply rudely because she isn’t feeding me.’ (C10, 17, M)

#### Subtheme 3.3: Poor growth support

Child welfare officers and shop-owners opined the reality for many of these children is that the parental figures, do not particularly care or plan the children’s life progression. All interviewed children expressed the desire to further their education but were sceptical of the parent’s support. Parental figures also expressed reservations about their ability to support their children’s academic progress or desires to pursue new directions:

‘Parents don’t care about the situation of these children. No plan, no savings.’ (WO8, 43, M)

‘Parents that has good plans for the child and wants a better future for the child will do fast to take the child off the street but this isn’t the situation for these children.’ (SO9, 45, F)

‘He already told me he wants to go into the business of trading. This news of him wanting to be a lawyer is strange to me [*laughs*].’ (PP5, 30, Mother)

A few of the children said if it is within the parents’ capability, the parent supports their wishes for new directions. Evident were few mothers, who part-sponsored vocational training like shoe-making (PC4, M, 17) and tailoring (PC7, M, 17) for their children:

‘I told my mum I want to be a soldier, so she took me to the army barrack and the soldiers promised that when I am done with junior secondary school, I can be admitted to army school.’ (PC3, 13, M)

#### Subtheme 3.4: Poor emotional connections

The interviewed children on the street did not care to discuss the topic of emotional connection with the parental figure; they flippantly gave ‘yes’ responses. However, some children and mothers elaborated on their lack of emotional connection. Shop owners and child welfare officers opined about a lack of affection and emotional connections between children on the street and their parents:

‘If I should carry my goods home, and I didn’t make enough sales, my parents will complain and blame me, aside from that we don’t talk much.’ (C1, 17, M)

‘He doesn’t tell me much. You know it’s what a child tells, I can know. (PP6, 52, Mother)

‘The fathers are usually not in the picture and they don’t have a good connection with the mothers. Some mothers get unnecessarily annoyed and beat the children when they don’t make enough money from the streets, not caring about the hazards.’ (WO9, 56, F)

Few mothers claimed they share a good bond with their children:

‘Once I notice that his mood is not right, I’ll call him and ask and he will tell me, what is bothering him.’ (PP4, 30, Mother)

#### Subtheme 3.5: Poor family bonding time

Most of the child welfare officers, shop owners, and some of the children said the norm for most families of children on the street is the inability to have quality family bonding time. Few children shared limitations to family bonding time because of the time needed to stay on the street for their economic activity:

‘They don’t have any family time, because some parents leave the home in the evening and stay out all night and the child go and comes as pleases.’ (WO11, 37, F)

‘I don’t hawk only on Sundays, that is the time we can bond. I tell them my thoughts sometimes but mostly I jot things in my diary.’ (C11, 17, M)

Only one parental figure shared his efforts at making provision for family bonding time others complained of time constraints:

‘I create enough time for my children to talk with them and for them to be free with me.’ (PP2, 40, Father)

## Discussion

The results showed that children on the street came from family structures characterised by broken homes and large-sized families. The family has poor economic and social resources, and challenges with children’s schooling or parental illiteracy. The relationship pattern between children on the street and the parental figure included poor adaptability, poor growth support, economic partnership with parents, poor emotional connection and lack of family bonding time. The three thematic results were intertwined, with relationships reflecting family resources premised on the broken family structure of children on the street of Ibadan, Nigeria.

### Broken homes and poor socio-economic resources of children on the street

The children in this study have structural family problems and attendant poor socio-economic family resources. A broken home is one in which a parent is absent, because of death or separation either formally (divorced) or informally.^7,21,22^ The patterns in this study included children living with unwedded mothers, and mothers who cohabited or married but got separated, divorced or widowed. These mothers lacked spousal support, and struggled with finances. Streetism for children who have existing links to their families has been attributed to structural family problems like broken homes as evident in this research.^21,22^ Mothers and fathers play significant roles in children’s well-being and neither of their roles can be neglected or substituted.^7^ It was not surprising that some children in this study complained about their absent fathers. The initial absence of some fathers was in search of better economic opportunities in other geographic locations. However, the fathers eventually withdraw from their first family, remarry or cohabit with another woman and start another family in another location. Supporting our finding, other researchers had reported the mobility of fathers in search of greener pastures in urban areas, making them migrate and re-settle.^23,24,25^ This engenders the acquisition of multiple female partners, with subsequent detachment and abandonment of the original ‘wives’ and their children with resultant broken homes, as revealed in this study.^23,24,25^ The studies further explained that broken homes exist because the housing set-up in urban areas does not allow several wives to live together and eventually ‘matricentric’ homes emerge, where mothers live with the children and the fathers are completely absent or arbitrarily visits.^25^

Poor spousal support for mothers was discussed at length by participants in this study. We found that most mothers and their children lacked both social and financial support, particularly from poorly committed unwedded fathers. A hypothesis proclaimed unmarried fathers tend to downplay their fatherly roles and are less materially and emotionally involved with the family compared to married fathers.^23,24^ This was the scenario in this study, which necessitated children taking to the street to support their mothers. Likewise, married fathers particularly those living separately from the family or in polygamous unions were materially and emotionally uncommitted to mothers and children on the street in this study. The necessity of joint parental responsibility for good child outcomes cannot be overemphasised.^23,24^ Streetism for some children in this study could have been averted if the families had adequate socio-economic resources. There was a mention of children on the street with socially inept young parents, who lacked parenting skills and were products of broken homes. The lack of an ideal parent role model for the immature parents of children on the street increases the odds of poor social support for these children. This underscores the need for school and community parenting programmes as suggested by other studies on street children because such programmes will entrench parenting responsibilities in all individuals from childhood and continue to adulthood.^26,27,28^

The economic resources of the parents were constrained because of low-cadre occupations and unemployment as found in this study and previously published in a Nigerian study as a solitary cause of child streetism.^9^ The parents of children on the street in this study were described as the ‘poorest of the poor’ who could not afford to provide a meal a day for themselves and the children. This is an interesting description but regardless of this, poor economic resources are known as ‘push and pull’ for child streetism, vastly cited in the literature.^9,29,30^ Children venture to the streets primarily to obtain some form of livelihood and make money for feeding because when a child is hungry nothing else appeals except to find means for obtaining food.^31^ This was one reason present and past children on the street of Ibadan citied for venturing to the street but over time they save up to support their other needs. In this study, working older siblings were willing to support the children on the street but were not gainfully employed, were apprentices, or have personal financial burdens, as previously reported.^32,33^ Although the economic situation in Nigeria is harsh and many families are struggling to manage the resources at their disposal,^8,9,10^ not all financially constrained parents have children on the street. It seemed as if child streetism is inevitable for families with structural problems, poor social resources in addition to poor economic resources.

The Child Right Acts (CRA) was adopted in 2003 in Nigeria but had not been enforced.^34,35^ Parents are the expected custodians of children’s rights and street hawking by children is a considered a form of child abuse, child labour and violation CRA,^34,35^ but Nigeria lacks commitment to the CRA and parents are not held accountable.

### Large family size and poor socio-economic resources of children on the street

This study revealed an association between large family size and the presence of children on the street. A large family size in Nigeria is considered when there are more than 5.0 persons per monogamous household, that is, more than 3.0 children per monogamous family.^35,36^ A large family size stretches the finances of the family with the need for children to take to the street and engage in economic activities to fend for themselves and support the family finances.^21,22^ In a large family, the cost of raising children can also overwhelm the financial resources with consequent child streetism. Large family size settings related to a family’s financial incapacity have been published as a major factor contributing to poor child care and push for child streetism.^21,22^

Worse child outcomes are associated with household chaos in instances of polygamy and large family sizes as discovered in this study.^21,22,25^ Although some participants suggested an association between polygamy and child streetism, other participants believed that the association of polygamous families with child streetism was not true. Polygamous and/or large families can be overridden by good parenting strategies which is a valid and proven idea.^21,32,33^ Responsible parenting can be defined as undisrupted nurturing of a child while maintaining balance warmth and control, with effective monitoring, non-coercive discipline and clear expectation.^26,27^

### Parental illiteracy and streetism as an educational resource for children

It was deduced from this study that poorly educated parents do not prioritise children’s schooling but rather send them to the streets to support family finances. This isn’t a novel finding, because it has been documented that parents who are poorly educated and financially incompetent preferably send the children to the street to supplement the family’s income.^10,37,38^

In the purview of poor economic resources of the parents in this study, streetism for some children served as an educational resource where they make money to support current schooling or save towards further education. These children experience interruptions in schooling, some with eventual school failure while some are outrightly withdrawn from school. These educational challenges have consequences and of particular concern are ‘social adaptation’ problems for these children in the future.^32,33^ It is however laudable that some mothers in this study, part-sponsored their children’s desire for academic growth or vocational training against the odds of poor financial capability and poor spousal support.

### Cultural ambivalence and child streetism

The parents’ desire for their children to acquire skills in the family trade, viewed as a culturally permissive idea, accounted for the presence of some of the children on the street of Ibadan as previously reported in Tamale Ghana.^38^ Although involving children in trading on the street is regarded as a violation of child’s rights in Nigeria.^6,10^ Parents view it as a learning process that can be a fallback for the children in the future should they encounter job or school failure.^38,39^

The loss of the collectivist culture of rearing children is a trend affecting families in sub-Sahara Africa, and this was also reported in this study as a cause of the continuing presence of children on the street of Ibadan.^25^ The modernisation of families has led to the loss of the traditional African kinship-oriented system and culture of collective child-raising.^25^ This is because the small urban-slum housing units of the children in this study do not allow for extended families cohabitation. Accordingly, a Nigerian study published a Yoruba adage saying ‘Only one father gives birth to a child but hundreds of other surrogate parents in the community are waiting outside there to train and bring up the child’ explaining the importance of collectivist culture.^40^ The study supports the need to take into consideration other persons in the child’s mesosystems that can halt the multiplication of children on the street, as previously suggested.^40^

The report of parental irresponsibility and devaluation of family life in favour of promiscuity was alluded to as a trademark of some Ibadan indigenes in this study. It was reported that promiscuity and serial cohabitation produce children who are uncared for and abandoned, with aged grandmothers who lack the resources and skills for effective parenting. Living with grandmothers encourages resilience for street-involved children but this is when grandmothers are supported socially and financially.^1,41^ Uprooting a child from a nuclear family to guardian care predisposes them to the risk of developing learned helplessness, psychological problems, and developmental delays, worse for the unmonitored child mingling with street elements.^42^ This is a concern for children on the street who are living with guardians; therefore, the culture of serial promiscuity with abandoned children should be addressed.

### Child streetism and spiritual backdrops

We found some parents committed their children to the street in the hope of divine intervention that can bring forth a positive outcome for the child. A study conducted among homeless street children reported they believed in God for a chance at a better life while living on the street.^39^ Structural problems and lack of resources in the families of street children have been linked to parents’ shift in worldview and distortion of reality.^43^ This may be the reason for the spiritual rationalisation of these parents in conjunction with their poor literacy level. The notion of spiritual belief that unplanned children will be sorted by God when they are sent out to the street by their parents needs to further research.

### Poor adaptability and poor family bonding time

A child usually receives more parenting from the resident parent, mostly the mother as was the case for the children in this research. The available parent is usually challenged by the time demand and socio-economic struggle of the lone residential parent.^7^ This may be the reason the children in this research could not elaborate on relationships with their mothers because time demands did not afford them family bonding. The children reported almost non-existent relationships with the unavailable fathers and when available the fathers hardly have time to bond or form a tangible relationship.

Creating family time for personalised dialogue between a child and the parents is the essence of a nurturing relationship, which affords an avenue to share and solve problems.^44^ Personalised dialogue between a child and parents has been proven to avert the effect of any ACEs (Adverse Childhood Experiences) and promote the healthy development and well-being of children.^44^ However, many children in this study reportedly bear and solve all their problems by themselves which depicts poor adaptability. Poor adaptability if unchecked can lead to the breakdown of the parent–child relationship and has been reported as a cause of children leaving the home permanently for the streets or elsewhere.^43,44^

### Poor parent–child emotional connections

The reports of poor parent–child emotional connections and the unwillingness of children on the street to elaborate when probed about the display of affection with their parents connote limited or strained interactions. The lack of filial emotional connection is concerning because poor filial emotional connection and display of affection are precursors for antisocial behaviour in adulthood.^45^ Lack of parental nurturing documented in this study in the background of poor economic resources and school disruptions can result in poor formal and informal support for the children which can mediate negative psychosocial problems for the children in the present and future.^42^

### Economic oriented partnership

The need for economic partnership with parents imposes parentification on these children.^46^ Parentification is when a child assumes parental roles of financial provider and together with emotional disconnect the children are at risk of behavioural problems in adolescence and antisocial behaviour in adulthood.^45,46^ The ‘power of earning money’ whereby the child feels he or she is equal to the parental figure and does not feel the need to share decision-making with the parent was evident in this study. This is another dimension of parentification that negatively influenced the desire for new directions of children on the street because they do not care to discuss their plan for growth with their parents or were sceptical.

The reported inability of most children on the street to form a genuine partnership with their parents aside from the ‘economic interest partnership’ is worrisome. It was gathered that parental figures were generally unconcerned about the child’s situation on the street, and the child’s movements and they don’t have any plan for the child’s progression except to obtain monetary returns. This is in keeping with a Nigerian publication of the *laissez faire* parenting style among families of street children in general which lacked specifics for children on the street.^47^ Noteworthy in this research is the empathy towards the mother’s poor financial situation and parentification of children on the street, particularly boys who feel obliged to assist the mother when the father does not meet up to expectations.

A 2015 study found that enabling parent–child interpersonal relationships can numb the effect of poor economic and social resources with a positive emotional and behavioural outcome for adolescents.^46^ This had been the position of the African Union since 1999 when they pronounced within the Charter on the Rights and Welfare of the Child that ‘a child should grow up in a family environment where there is happiness, love and understanding’.^48^ The cumulative effect of structural family problems, poor family resources and poor filial relationships on the socio-emotional outcome of children and adolescents has been generating research interest in recent times. This exploration of the family dynamics of children on the street has filled some gaps in this regard.

### Strength and limitation

The sampling strategy and inclusion of paired parent–child IDIs are strengths of the study because it afforded the richness of information derived and family-level insight. The teenage inclusion criterion for the children on the street was selected because of ethical considerations and could be viewed as a limitation because the voices of younger children were not captured.

## Conclusion

Based on the results of this research, parental literacy, employment, understanding of effective parenting and children’s rights, regardless of parents’ differences and situation, spiritual or cultural beliefs could be important foci in further research or strategies to reduce children on the street.

### Recommendation

Strengthening family resources can help parents realise their roles in protecting children’s rights. Community leaders could be involved in identifying families that need help regarding a child’s well-being and they should notify the states’ welfare service. Family-level interventions to curb the epidemic of children on the street of Southwest Nigeria is a suggestion for further research.
